# Training needs for Ugandan primary care health workers in management of respiratory diseases: a cross sectional survey

**DOI:** 10.1186/s12913-020-05135-3

**Published:** 2020-05-11

**Authors:** Rebecca Nantanda, Gerald Kayingo, Rupert Jones, Frederik van Gemert, Bruce J. Kirenga

**Affiliations:** 1 Makerere University Lung Institute, Makerere University College of Health Sciences, Kampala, Uganda; 2 University of California Davis, Davis, CA USA; 3 Peninsula Medical School, Plymouth University, Plymouth, UK; 4 Groningen Research Institute for Asthma and COPD (GRIAC), University of Groningen, Groningen, The Netherlands; 5 Department of Internal Medicine, Makerere University College of Health Sciences, Kampala, Uganda

**Keywords:** Respiratory diseases, Primary care, Health workers, Knowledge, Skills

## Abstract

**Background:**

Respiratory diseases are among the leading causes of morbidity and mortality in Uganda, but there is little attention and capacity for management of chronic respiratory diseases in the health programmes. This survey assessed gaps in knowledge and skills among healthcare workers in managing respiratory illnesses.

**Methods:**

A cross sectional study was conducted among primary care health workers, specialist physicians and healthcare planners to assess gaps in knowledge and skills and, training needs in managing respiratory illnesses. The perspectives of patients with respiratory diseases were also sought. Data were collected using questionnaires, patient panel discussions and review of pre-service training curricula for clinicians and nurses. Survey Monkey was used to collect data and descriptive statistical analysis was undertaken for quantitative data, while thematic content analysis techniques were utilized to analyze qualitative data.

**Results:**

A total of 104 respondents participated in the survey and of these, 76.9% (80/104) were primary care health workers, 16.3% (17/104) specialist clinicians and 6.7% (7/104) healthcare planners. Over 90% of the respondents indicated that more than half of the patients in their clinics presented with respiratory symptoms. More than half (52%) of the primary care health workers were not comfortable in managing chronic respiratory diseases like asthma and COPD. Only 4% of them were comfortable performing procedures like pulse oximetry, nebulization, and interpreting x-rays. Majority (75%) of the primary care health workers had received in-service training but only 4% of the sessions focused on respiratory diseases. The pre-service training curricula included a wide scope of respiratory diseases, but the actual training had not sufficiently prepared health workers to manage respiratory diseases. The patients were unsatisfied with the care in primary care and reported that they were often treated for the wrong illnesses.

**Conclusions:**

Respiratory illnesses contribute significantly to the burden of diseases in primary care facilities in Uganda. Management of patients with respiratory diseases remains a challenge partially because of inadequate knowledge and skills of the primary care health workers. A training programme to improve the competences of health workers in respiratory medicine is highly recommended.

## Background

Globally, respiratory diseases are among the leading causes of death in both adults and children. About 90% of the deaths from respiratory diseases occur in low and middle-income countries (LMICs) [1] The World Health Organization (WHO) estimates that by 2030, lung diseases will account for about one in five deaths worldwide [2]. Currently, Chronic Obstructive Pulmonary Disease (COPD) is the third leading cause of death and it is projected that COPD will cause more deaths than HIV, Malaria and Tuberculosis all put together [3, 4]. Generally, LMICs are disproportionately affected due to the double burden of communicable and non-communicable respiratory diseases [5]. For example, of the 235 M people with asthma worldwide, 50 M are from Africa. More than 80% of the asthma deaths occur in LMICs like Uganda, usually related to lack of proper treatment [4, 5]. Moreover, the mortality and morbidity due to asthma and COPD may be underestimated as many are underdiagnosed and treated for respiratory infections [6–11].

Like many countries in sub-Saharan Africa, Uganda is experiencing an emerging yet under-recognized epidemic of non-communicable respiratory diseases [12, 13]. According to the Ministry of Health Non-Communicable Diseases (NCD) morbidity and mortality assessment, an estimated 15% of men and 8% of women with NCD die from chronic respiratory diseases. A study in Masindi district (Western region of Uganda) showed a high prevalence of COPD at 16.2% (15.4% in men, 16.8% women), and many of them were unaware about COPD, including risk factors such as biomass and tobacco smoke [14, 15]. A recent national survey on asthma among persons 12 years and older revealed a prevalence of 11.02%, and the majority (98%) of them were undiagnosed and untreated [16]. According to the Uganda Registry for Asthma and COPD (URAC), 60.3% of Ugandan asthmatics experienced ≥3 attacks in 1 year and asthma mortality is 27.3 per 1000-person years [17]. Children are also affected by chronic respiratory diseases. A study among children less than 5 years of age with acute respiratory symptoms showed that up to 41% were found to have asthma syndrome [9].

Despite this high burden of respiratory diseases, there has been little attention and limited human resource capacity to manage these illnesses [18]. Until recently, most of the health programs on lung health focused on communicable diseases such as acute respiratory infections (ARI), pneumonia and tuberculosis. Little has been done about chronic respiratory diseases such as asthma, COPD and lung cancer [19]. There is need for capacity building such as retraining frontline health workers to screen, manage and appropriately refer patients who present with respiratory illnesses. There is also need for increased advocacy about lung health among policy makers.

This study aimed to assess the level of knowledge and skills of frontline health care workers in managing respiratory illnesses. In addition, we sought the opinions of the frontline healthcare workers, specialist clinicians and healthcare planners about the proposed in-service training programme on respiratory medicine for primary care health workers.

## Methods

### Study design and setting

This was a cross sectional study involving health workers in primary care settings, specialist clinicians and health care planners. The health workers included general doctors, clinical officers, nurses and midwives. In Uganda, general doctors have training in medicine and general surgery for 5 years but have no specialization. Clinical Officers under-go a three-year diploma course in clinical medicine. Although nurses and midwives traditionally provide nursing and midwifery care respectively, the shortage of staff in Uganda has led to task-shifting and many of them act as clinicians in their health facilities. The specialist clinicians included pulmonologists, internists and pediatricians, while health care planners were heads of hospitals and district health services.

The health care delivery system in Uganda comprises of 6 levels; 1) Health Centre II which is the lowest level and provides basic outpatient clinical care, 2) Health Centre III where there are maternity services in addition to outpatient clinical care, 3) Health Centre IV where there are both out- and in-patient facilities as well as emergency obstetric care, 4) District hospital which offers services similar to HC IV at a larger scale in addition to other services such as general surgery and dental services and has a wider range of diagnostics such as Chest X-ray and laboratory services, 5) Regional referral hospital which offers services similar to those of a district hospital and specialized care and 6) National referral hospital which offers specialized and super-specialized services. The primary care services are offered from HC II to district hospital. However, in some cases, patients report to the regional and national referral hospitals for primary care services. Patients that cannot be managed at one level of health care are referred to the next level of care.

### Development of the survey questionnaire

The survey questions (Additional files 1, 2 and 3) were developed through a series of discussions by experts including physicians (internists and pediatricians), pulmonologists, primary care physicians and medical educators with experience in pre-service and in-servicing training. The questions were designed to cover a wide range of aspects that are critical in disease prevention and management, and health promotion which are core functions of primary health care. The questionnaire for the primary care workers focused on; burden of respiratory diseases in the health facilities based on healthcare providers perception, assessing the current level of knowledge and skills of the primary care health workers and identifying the knowledge gaps, identifying the training needs of the primary care health workers, organization of the services at the health facilities, availability of medicines, equipment, supplies, and reference resources for respiratory care, individual and health system issues that hinder effective service delivery and, opportunities for continuous professional development. In addition, the respondents were asked to make suggestions on the best model for delivery of an in-service respiratory medicine training programme and the anticipated personal and health system benefits.

The questionnaire for specialist clinicians and health care planners contained items about burden of respiratory diseases, training content needed for the primary care clinicians to provide high quality of care to patients, challenges in patient care and the anticipated benefits of the training to the patients, themselves (specialists and healthcare planners) and the health system.

The questionnaires were pre-tested on 20 participants from rural and urban-based primary care facilities, physicians, and health care planners, to check for understanding and relevance of content, and make suggestions for improvements.

### Participants

The participants were drawn from different regions of Uganda, and from each of the different levels of care. They were grouped into four categories:

Clinicians who provide day-to-day clinical care in the primary care facilities and included doctors, clinical officers, nurses and midwives. These were collectively referred to as primary care clinicians because they are the first point of contact for the patients seeking for care. They shared their views about respiratory health care services in their workplaces.
Specialist clinicians who included Pulmonologists, Internists, and Paediatricians were included in the study to share their experiences in managing patients with respiratory symptoms who had been referred by primary care clinicians or were seen by them as their (patients) first point of contact with the health care system. They also shared their perspectives on the improvements that would be needed to improve the care of patients with respiratory symptoms in primary care settings.
3) Health Care Planners who included mainly hospital administrators and District Health Officers
Patients with chronic respiratory diseases and were attending the chest clinics of the national referral hospital.

### Sampling process

A variety of methods were used to identify and reach out to potential participants.

- District Health Officers and Hospital Administrators: We reached out to the heads of the district health services and hospitals either through e-mail, social media, telephone calls or physical visits. They were also able to provide information about how to access the nurses, midwives and clinicians in their areas of jurisdiction.
- The Ministry of Health register for doctors, and the register for Uganda Medical Association (a doctors’ association) were also used to identify potential participants. Using these registers, we were able to identify specialists in Internal medicine and Paediatrics as well as the general doctors, who were our potential respondents. E-mails and social media were used to reach out to potential respondents
- Patients with chronic respiratory diseases were identified through the weekly chest clinics at Mulago national referral hospital

### Data collection

The data were collected using quantitative and qualitative methods. Quantitative data was collected using the survey questionnaires. The questionnaires were sent out to potential respondents (as described above) using e-mail with a link to an online software programme (Survey Monkey). Each of the three categories of respondents (primary care health workers, specialist clinicians and health care planners) had specific questionnaires applicable to their respective roles in health services delivery. Respondents who did not have access to computers and/or internet were given hard copies of the questionnaires which were self-administered and returned to the survey team. In such cases, the data were then manually entered into the Survey Monkey software programme by an independent data entry clerk.

We also looked at the training curricula for doctors, clinical officers, midwives and nurses, to check for the topics related to lung health and the amount of time allocated to them, to get an idea on the scope and intensity of training that is provided to pre-service training with respect to respiratory health. The Ministry of Health policy documents such as the Health Sector Development Plan and National Health Policy were also checked to get an insight into the attention paid to respiratory health at national level.

Qualitative data was obtained through discussions with two patient groups, to collect information about their experiences during their initial consultations for respiratory symptoms and suggestions for improvements in quality of care. These discussions were held as part of the routine health education sessions within the hospital.

### Data management

Quantitative data was analyzed using descriptive statistics. The information from the curricula were analyzed using specific themes of interest such as range of topics covered under respiratory medicine, chronic care approaches, organization of the teaching (practicals versus theory), among others. The policy documents from the ministry of health were reviewed to determine the extent to which respiratory diseases are part of the national priorities. Thematic content analysis techniques were utilized to analyze qualitative data.

### Ethical considerations

The data for this manuscript were derived from a Training Needs Assessment aimed at informing the development of a training programme on Respiratory Medicine for primary health care workers. The project was therefore categorized as a Quality Improvement project. In addition, we consulted the Institutional Review Board (IRB) from University of California Davis where one of the authors (GK) is a staff member for their opinion regarding the ethics, and it was determined that the survey was basically part of a quality improvement project and therefore did not require formal IRB review.

Consent to participate in the survey was implied by responding to the survey questions. Using the Survey Monkey system, it was not possible to know who the respondent was, and hence confidentiality was in-built. The respondents completing hard copies of the questionnaires were not required to provide any identifying information and researchers were unaware of the source of the data eg hospital or clinic. Data about the patients’ perspectives was collected during health education sessions at the chest clinic of Mulago national referral hospital. Participants were given health education as routinely done and during the discussions, they were asked about their experiences during their first consultations for respiratory symptoms and suggestions for improvement.

## Results

Overall, there were 104 respondents; 80 (76.9%) were primary care health workers, 17 (16.3%) specialists and 7 (6.7%) health care planners. Among the primary care workers, majority were clinical officers (47.3%) followed by registered nurses (18.8%). The details of the demographic characteristics of the respondents are provided in Table 1. It was not possible to ascertain the response rate because various methods were used to seek respondents including social media.

**Table 1:** Demographic characteristics of respondents (*N* = 104)

Characteristic		Number (n)	Percentage (%)
Gender	Male	58	55.8
	Female	45	43.3
	Gender not indicated	1	0.9
Specialist Clinicians (*n* = 17)	Pulmonologists	2	11.8
	Internists	11	64.7
	Pediatricians	1	5.9
	*Other	3	17.6
Primary Care Clinicians (*n* = 80)	Medical officers	3	3.8
	Clinical officers	36	45.0
	Midwife (degree level)	1	1.3.0
	Registered Nurse	15	18.8
	Enrolled Nurse	10	12.5
	Enrolled midwife	3	3.8
	*Other	7	9.5
Health Care Planners (*n* = 7)	District Health Officers	3	42.9
	Hospital Directors	1	14.2
	Head of Department in academic institutions	3	42.9
Total		104	

*Others included Public Health Specialists, District Health Officers, Orthopedic assistants, Radiographers and Nursing assistants

### Burden of respiratory diseases in primary care facilities

Over 90% of the clinicians (primary care health workers and specialists) reported that they frequently see patients with respiratory diseases during their day-to-day work, with majority (58%) of them indicating that at least 6 of every 10 patients present with respiratory symptoms. Similarly, the health care planners indicated that many of the patients that present in the health facilities have respiratory symptoms. Non-communicable chronic respiratory diseases such as asthma were among the top five diseases seen in primary care settings. Interestingly, COPD, which is one of the common chronic respiratory diseases and contributing significantly to mortality, was not mentioned among the five most common diseases indicating likely under-diagnosis.

### Knowledge and skills of primary care health workers

**Fig. 1:**
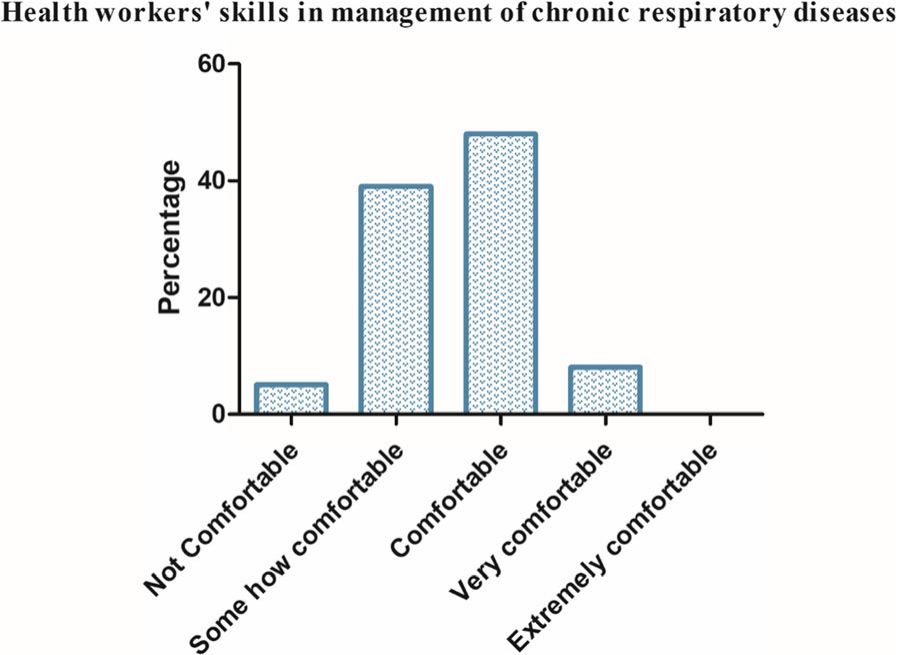
Knowledge and skills of health workers in managing respiratory diseases

When the primary care health workers were asked about how comfortable they were in diagnosing and managing respiratory diseases, only 8% indicated that they were very comfortable in doing so. More than half (52%) of the respondents indicated that they were not comfortable in diagnosing and managing respiratory diseases (Fig. 1). The greatest challenges were for chronic respiratory diseases particularly COPD and asthma.

Similarly, very few primary care health workers (4%) indicated that they were comfortable to perform common procedures for diagnosis and management of respiratory conditions like pulse oximetry, nebulization, giving oxygen, performing and interpreting Mantoux test, interpreting spirometry results, demonstrating how to use a spacer and measuring Peak Expiratory Flow Rate (PEFR).

### Opportunities for in-service training

The majority (75%) of the primary care health workers had received in-service training in the 3 years prior to the survey. However, only 4% of the respondents had received any form of in-service training on chronic respiratory diseases. Specifically, none of the respondents had received any training in COPD or lung cancer screening. Interestingly, more than half of the respondents had received training in communicable respiratory diseases such as tuberculosis.

When the respondents were asked about training in other competences which are critical in improving quality of care, such as chronic care, team-based care, communication and patient-centered care, only 5.5% had received training in any one of these areas.

The responses of the health care planners correlated with those of the health workers, with 71% of them indicating that they had provided in-service training opportunities for their staff, but none of them focused on chronic respiratory diseases.

### Referral practices for patients with respiratory diseases

The findings showed that the patients that primary care health workers commonly refer to specialists are quite diverse, with both communicable and non-communicable diseases such as TB (15.3%), pneumonia (10.5%), COPD (46%), asthma (14.4%) and bronchiectasis (32.8%). However, when the specialists were asked about the type of patients that are referred to them, 72% of them indicated that many of the referrals from primary care were unnecessary because they could be managed at that level. Specifically, the specialist indicated that majority of the patients with asthma could be managed in primary care settings if the health workers were given the necessary competencies.

### Barriers to care of patients with respiratory illnesses

The major barriers to provision of quality care to patients with respiratory illnesses were pervasive and diverse, and included: inadequate knowledge and skill of health workers, inadequate diagnostics, drugs and equipment, lack of opportunities for in-service training, inadequate staffing, inadequate supervision and heavy workload. Similar views were echoed by the healthcare planners and specialist clinicians.

### Pre-service training programmes

The primary care health workers were asked about their views on the pre-service training they received from the training institutions and how much it prepared them for the clinical care that they are expected to provide. More than half (58%) indicated that their basic training was insufficient, and that they needed further training to be able to competently manage the patients with respiratory diseases.

### In-service respiratory medicine training

**Fig. 2:**
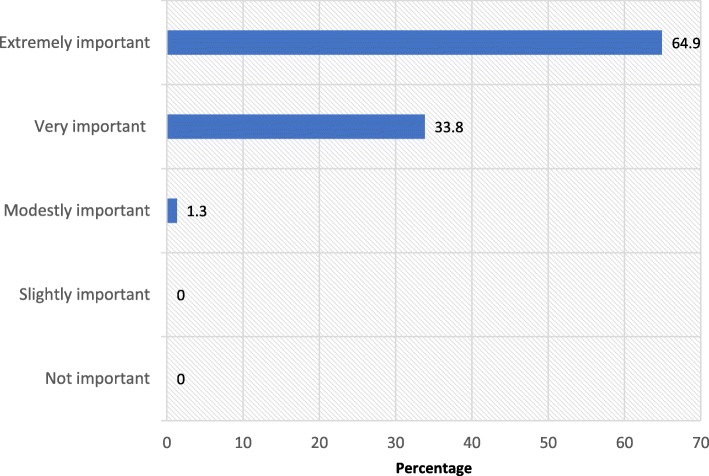
Importance of the respiratory medicine in-service training programme

All the three categories of respondents (primary care health workers, specialist clinicians and health care planners) indicated that the training of primary care health workers in respiratory medicine was important, with more than half of them indicating that it was extremely important (Fig. 2).

Furthermore, the health workers indicated that they needed training in majority of the respiratory diseases and procedures, with the following areas being ranked highest; COPD, Asthma, bronchitis, pneumonia, reading and interpreting chest x-rays and administering inhaled medicines.

The specialist clinicians also resonated with the primary care health workers by indicating that the training would be extremely beneficial, not only to the primary care clinicians but also to the specialists. Such benefits included; reduction in unnecessary referrals and hence workload, fewer patients who are referred late, and improvement in patient outcomes.

The specialists further highlighted specific areas which they thought the primary care workers needed training; clinical evaluation skills, interpretation of imaging tests, spirometry, respiratory pharmacology, recognizing emergencies and providing pre-referral care, non-pharmacological treatment such as nutrition and chronic care. In addition to the above areas, the policy makers suggested training in disease prevention, patient education and appropriate referrals for patients with respiratory diseases.

The respondents further suggested that a hybrid model of the training programme would be the best option for them. This would involve 3–5 days of course work at Makerere University Lung Institute, followed by self-directed learning at their work stations, supported by mentorship visits from the course instructors.

### Findings from pre-service training curricula

- *Nurses and midwives:* The curriculum for nursing and midwifery pre-service training largely focuses on their primary roles of nursing and midwifery, with very minimal coverage on clinical aspects addressing diagnosis and management of disease conditions. For example, only about 1.3% of the entire 4-years Bachelor of Science in Nursing curriculum has some elements on lung health care
- *Clinical officers:* The curriculum for clinical officers showed that during their three-year course, they are expected to cover both the theoretical and practical aspects of medical care. The scope of the training includes; history taking and performing a general and systemic examination, diagnosis of medical conditions, and interpreting laboratory and radiological results. Regarding respiratory conditions, the curriculum covers history taking, general and respiratory examination, diagnosis and management of respiratory diseases such as pneumonia, tuberculosis, asthma, bronchiolitis, COPD, Pleurisy/pleural effusion and suppurative lung diseases (bronchiectasis, lung abscess and empyema thoracis), and the relevant equipment for management of these conditions. In addition, there are course units in applied microbiology, pathology and pharmacology of the respiratory system. To a large extent, the emphasis is acute and tropical respiratory illness
- *Medical officers:* The training for medical officers covers a wide range of aspects that would be necessary for the management of patients with respiratory symptoms. They include basic science in the physiology, anatomy, pathology, pharmacology and microbiology of respiratory system. In addition, they are trained in practical skills of history taking, physical examination, investigations and management of patients. The curriculum also covers important aspects of patient care such as communication, health systems management and chronic care.

### Patients’ experiences and views about quality of care

When patients were asked about their experiences during the first visit to a primary care facility for respiratory complaints, many of them indicated that they were not satisfied with the outcome. Some of them were diagnosed with malaria and given antimalarial medicines despite having expressed their respiratory symptoms, while others thought the consultation was very brief and inadequate, and that the medicines they were given did not help them at all. Many patients were not informed about their diagnosis, and were only given medicines, some of which they had used before visiting the health facility. Multiple visits to the health facilities before arriving at a diagnosis were also reported and this was noted to have been a strain to their financial resources, time and was quite frustrating. It was not until they were referred to the specialists that they were able to understand the diagnosis and get the right treatment. These observations were irrespective of the type or cadre of primary care provider seen before going to the specialist.

## Discussion

This study aimed to understand the current practices in diagnosis and management of patients with respiratory illnesses in primary care health facilities, identify gaps, and seek suggestions on how to make improvements. Overall, the findings indicated that respiratory diseases are a major burden to all sectors of the health services in Uganda. However, there were major gaps in the training offered and knowledge, skills, competences and facilities for the diagnosis and management of patients presenting with respiratory symptoms. Lastly, the primary care health workers, specialists, healthcare planners and patients indicated that the identified gaps could be mitigated by provision of focused in-service training to primary care health workers. The survey also revealed that COPD was not highlighted among the top five diagnoses for respiratory symptoms. This is despite evidence that COPD is one of the leading chronic respiratory diseases in LMICs [20]. These findings confirm the results from a similar study in Masindi district-Uganda which revealed that health workers had very low awareness of COPD. The Global Burden of Disease study also shows that in Uganda, COPD is the ninth biggest cause of years lived with disability [21]. Studies elsewhere have also highlighted the low knowledge of health workers on COPD [8, 11]. Consequently, patients with COPD are missed, and therefore do not receive appropriate and timely care. Clearly, there is need for capacity building of the primary care health workers to improve their knowledge, skills and competencies to manage patients presenting with respiratory symptoms, especially in the prevailing face of task-shifting [22, 23]. In high income countries, the recognition of the central role of primary care in the identification, diagnosis and structured management of chronic disorders has been addressed using training roles and management systems to monitor long term condition care [24, 25].

Our findings further indicated that none of the Continuous Professional Development (CPD) opportunities are focused on chronic respiratory diseases, even though they contribute significantly to the burden of disease in the health facilities and communities. This may be related to low awareness of the impact of respiratory diseases on the overall quality of life of the affected patients among the health workers and the healthcare planners who are responsible for providing CPD opportunities. In fact, COPD is not even mentioned among the priority diseases by the Government of Uganda Health Sector Development Plan [19]. There is need to increase awareness about respiratory illnesses among health workers, policy makers and general population.

Another key finding was that clinical officers and nurses constitute most of primary health care providers. Although the pre-service training curriculum for clinical officers seemed enough to provide knowledge on respiratory diseases, they indicated that they had challenges in managing patients with respiratory symptoms. This may be partly due to the method of delivery of the courses during pre-service training or the general loss of knowledge and skills over time. It could also be about the depth of coverage. In general, clinical officers conduct their clerkships in clinics that see mostly patients with communicable diseases. There is no mandate to cover non- communicable diseases prior graduation. It is likely that COPD is covered during the didactic portion but not sufficiently covered during the practical portion of the training. On the other hand, the pre-service training for nurses and midwives does not address majority of the roles and responsibilities in clinical care that they are assigned to perform in the health facilities. Therefore, all cadres of frontline healthcare providers could benefit from a refresher training program to complement the pre-service training. This is critical because task-shifting is a recognized and well documented strategy for health service delivery in Uganda [23, 26] and the involved persons need to be well equipped to effectively deliver in their new roles, which are clearly beyond their pre-service training. The health care providers, specialist clinicians and healthcare planners indicated that an in-service training programme in respiratory medicine would be very important. Additionally, they indicated areas with big knowledge gaps and proposed a course delivery model that would work for them. These revelations are a great motivation to addressing the gaps in care of patients with respiratory complaints, and one of the solutions is an in-service training programme in respiratory medicine.

Considering the findings that respiratory diseases contribute to the greatest burden of disease in primary care health facilities in Uganda, it is of great concern that most of primary care health workers do not feel comfortably knowledgeable and skilled to manage patients under their care. This is in sharp contrast to their day-to-day activities which involve clinical care (diagnosis and management) and task-shifting. In Uganda, most of the patients in rural settings are seen by clinical officers or nurses for primary care, regardless of the level of health facility. Therefore, the low levels of knowledge and skills of these primary care providers in managing patients with respiratory diseases is of great concern, because it may be partly contributing to poor patient outcomes including avoidable complications and deaths, and high health care costs to the individuals and health systems, arising from repeated consultations with less competent health workers. Although this study did not collect data on health economic assessment that arises from the repeated consultations and ineffective treatments arising from the low competencies of the clinicians, it is possible that these processes were associated with avoidable and unnecessary personal and health system expenditure which could have been avoided if the primary care clinicians were competent. Indeed, studies show that major financial savings occur with better community access to diagnostic and effective clinical care [27, 28].

Lastly, the primary care health workers, specialists, healthcare planners and patients indicated that the identified gaps could be mitigated by provision of focused in-service training to primary care health workers. Another aspect that does affect the management of asthma and COPD regards the frequent co-morbidity with other long-term conditions such as cardiovascular diseases [29–31].. Although the issue of co-morbidity was outside the scope of this study, it is expected that the rising incidences of non-communicable diseases in Uganda will complicate the diagnosis and management of asthma and COPD [18]. In addition to gaps in knowledge and skills, Ugandan primary care is hampered by lack of continuity in the diagnosing of chronic respiratory problems as well as by lack of skills building of technological diagnostic procedures. Studies from other countries have also shown gaps in knowledge and skills among primary care clinicians for managing patients presenting with chronic respiratory symptoms [32]. For example, Yawn, et al. 2008 found that primary care physicians, Nurse Practitioners (NPs), and Physician Assistants (PAs) working in some parts of the US lacked awareness of COPD guidelines [33], and a similar problem was detected in the UK amongst practice nurses many of whom lacked confidence and training [34]. However, knowledge and skills for managing chronic respiratory disorders by primary care clinicians can be improved through effective on-job training [35]. Indeed, well trained nurses and other non-physician clinician have been shown to play a critical role in managing chronic diseases such as asthma and COPD [36–39].

### Methodological considerations

The study involved a variety of respondents who are involved in the management of patients in primary care such as medical doctors, clinical officers, nurses and midwives, both in rural and urban settings. The views of specialist clinicians, healthcare planners and patients were also sought, and they correlated with those of the primary care health care workers. The approach of involving multiple stakeholders in the survey strengthens the validity of the data.

It was not possible to compute the participant response rate because of use of many recruitment channels including social media, which may have impacted on the representativeness of the data. However, the health professional background of the respondents is in proportion to the distribution of background disciplines in Uganda.

There could have been a bias in estimation of the burden of respiratory diseases since this was based on their recall and perceptions, with no specific measurements done. However, the errors arising from this approach is likely to be very small because studies have shown that clinicians’ estimates of burden of disease usually correlate with records [40].

Lastly, it is possible that the people who responded were those with keen interest in respiratory illnesses thus creating self-selection bias.

## Conclusions

Respiratory illnesses contribute significantly to the burden of diseases in primary care health facilities in Uganda. However, the capacity of health workers to manage patients with respiratory diseases is low partly because of inadequate knowledge and skills. Opportunities to improve the health workers’ competences are limited due to lack of in-service training programmes and continuous professional development sessions with a focus on respiratory medicine within the health care system. Continuing education such as a respiratory medicine training programme is highly recommended. Blended learning using a combination of E- learning resources and practical session could also be helpful. Guidelines for management of respiratory diseases to be developed and availed as reference materials in primary care settings. This would contribute to better competencies of health workers in prevention and management of respiratory diseases, and promotion of lung health. This will ultimately improve the health of the general population in Uganda.

Uganda faces a perfect storm of a rising burden of chronic respiratory diseases, a primary care workforce inadequately trained and equipped to detect and treat disease and prevent morbidity and mortality. Patients consequently face an unnecessary burden of symptoms and risk of dying. Poorly managed respiratory disease puts a huge pressure on patients, hospitals and the economy. The first and crucial step in breaking this vicious circle is an investment in training for new and existing staff. This approach has been successful in many western countries where the ageing population is experiencing an epidemiological shift from infectious diseases towards chronic respiratory morbidity.

## Supplementary information

**Additional file 1.** Survey tool for Health Care Providers: Questinnaire items for Primary Care Health Workers on knowledge, skills and competencies about management of respiratory diseases

**Additional file 2.** Survey tool for Specialists: Questinnaire on the views of specialists on the need for an Integrated Respiratory Medicine Training Programme for Primary Care Health Workers in Uganda

**Additional file 3.** Survey tool for Policy Makers and Administrators: Questinnaire on the views of on the Policy Makers and Administrators ed. for an Integrated Respiratory Medicine Training Programme for Primary Care Health Workers in Uganda

## Data Availability

The datasets used and/or analyzed during the current study are available from the corresponding author on reasonable request.

## Abbreviations

ARI: Acute Respiratory Infections
COPD: Chronic Obstructive Pulmonary Disease
CPD: Continuous Professional Development
CRD: Chronic Respiratory Diseases
HC: Health Centre
HIV: Human Immunodeficiency Virus
LMICs: Low and Middle-Income Countries
NCDs: Non-Communicable Diseases
URAC: Uganda Registry for Asthma and COPD
WHO: World Health Organization
