# Supplementary material for: Training needs for Ugandan primary care health workers in management of respiratory diseases: a cross sectional survey
Source: BMC Health Serv Res. 2020 May 11;20:402. doi: 10.1186/s12913-020-05135-3 (PMC7212561; doi:10.1186/s12913-020-05135-3)
Supplement: Supplementary file 1 — Additional file 1. Survey tool for Health Care Providers: Questinnaire items for Primary Care Health Workers on knowledge, skills and competencies about management of respiratory diseases [file 12913_2020_5135_MOESM1_ESM.docx]

**INTEGRATED RESPIRATORY MEDICINE TRAINING PROGRAMME FOR HEALTHWORKERS IN PRIMARY CARE SETTINGS (iBreath project)**

**MAKERERE UNIVERSITY LUNG INSTITUTE COLLEGE OF HEALTH SCIENCES, MAKERERE UNIVERSITY**

**Needs Assessment Tool for Health Care Providers**

In Uganda, , respiratory diseases are among the leading causes of death in both children and adults. Until recently, much of the health programmes on lung health focus on communicable diseases such as acute respiratory infections (ARI), pneumonia and tuberculosis. However, there is an emerging yet under-recognized epidemic of non-communicable lung diseases such as asthma, Chronic Obstructive Pulmonary Disease (COPD) and Lung Cancer. The combination of the communicable and non-communicable lung epidemics is a major threat to public health. Unfortunately, there are very few primary care HWs, with the knowledge and skills to effectively manage these epidemics. In recognition of these challenges, Makerere University Lung Institute (MLI) is planning to start an integrated respiratory medicine programme for frontline health care providers.

As a health care provider your views on whether this programme is needed, what it should cover and how it should be organized are important. We are therefore seeking your opinion by participating in this needs assessment survey. Your participation in this survey is voluntary, and the information obtained will be kept anonymous and confidential.

This questionnaire comprises two sections (A and B) for assessing your training needs as regards respiratory medicine. Please answer all questions as honestly as possible to enable us get a good idea of your training need. Read and follow the instructions under each section carefully.

**SECTION A**

- 1. **Demographics**
  2. **Sex** Male Female
  3. **Cadre**

| Medical officer |  | Degree midwife |  |
| --- | --- | --- | --- |
| Clinical officer |  | Registered midwife |  |
| Degree Nurse |  | Enrolled midwife |  |
| Registered Nurse |  | Other (Specify) |  |
| Enrolled Nurse |  |  |  |

- 1. Number of years in service ……………………..
  2. Level of your health facility

District hospital Health centre IV Health centre III Health centre II

**2.0. Burden of respiratory diseases in the health facilities**

In order to provide training that is relevant to population needs, we need to understand the current burden of respiratory diseases in the various health care facilities.

- 1. On a scale of 1-5, how often do you see patients who present with respiratory symptoms?

1Never 2  occasionally 3 sometimes 4  most of the time 5 all the time

2.2. Of all the patients that you see in a day, what percentage present with respiratory symptoms?

<20% 20-40%  40-60%  60-80%  >80%

2.3. Of all the patients that present at your facility/unit with respiratory complaints, which category constitutes the majority?

1. Children
2. Adults
3. Pregnant women
4. Elderly
   1. Below is a list of the common respiratory diagnoses. Choose the top five diagnoses in your facility, starting with the most common to the least common (1=most common and 5=least common).

| Acute respiratory infections (ARI) |  | Chronic bronchitis |  | Foreign body aspiration |  |
| --- | --- | --- | --- | --- | --- |
| Upper respiratory Tract Infections (URTI) |  | Asthma |  | Bronchiectasis |  |
| Lower Respiratory Tract Infections (LRTI) |  | Lung cancer |  | Pulmonary embolism |  |
| Pneumonia |  | Chronic Obstructive Pulmonary Disease |  | Acute respiratory distress syndrome |  |
| Tuberculosis |  | Bronchiolitis |  | Pulmonary hypertension |  |
| Acute bronchitis |  | Pneumothorax |  | Others (specify) |  |

**3.0. In-service training**

3.1. In the last three (3) years, have you had any in-service training in the following areas? (Check all that apply)

| 1 | Common infectious diseases e.g. malaria, HIV |  |
| --- | --- | --- |
| 2 | Non-communicable diseases e.g. hypertension, diabetes |  |
| 3 | Communicable respiratory diseases e.g. Tuberculosis, Pneumonia |  |
| 4 | Non-communicable respiratory diseases e.g. asthma, COPD |  |
| 5 | Maternal Health and newborn care |  |
| 6 | Quality of healthcare |  |
| 7 | Immunization |  |
| 8 | Quality improvement |  |
| 9 | Professionalism |  |
| 10 | Chronic disease management |  |
| 11 | Team-based approach to patient care |  |
| 12 | Patient-centered care |  |
| 13 | Diagnostics procedures for respiratory diseases |  |
| 14 | No in-service training at all |  |

3.2. In the last three (3) years, have you had any in-service training in any of the following respiratory diseases and related risk factors (Check all that apply)

| Asthma |  | Tobacco smoking |  |
| --- | --- | --- | --- |
| COPD |  | Tuberculosis |  |
| Screening for lung cancer |  | Air pollution |  |
| Pneumonia |  | Biomass smoke |  |
| Respiratory zoonosis |  | None of the above |  |

3.3. In the last three (3) years, have you participated in any Continuous Professional Development (Continuous Medical Education/Continuous Nursing Education) sessions at your facility?

Yes  No

**If Yes,**

3.4. Have you had CME sessions that focus on respiratory diseases?

Yes  No

3.5. What kind of learning resources do you have and have access to in your facility? (Check all that apply)

1. Library
2. Websites
3. Job aids focusing on respiratory diseases

**4.0. Knowledge and skills**

4.1. On a scale of 1-5, how comfortable are you in making a diagnosis in patients who present with respiratory symptoms?

1Not comfortable 2  somehow comfortable 3 comfortable 4  very comfortable 5 extremely comfortable

4.2. On a scale of 1-5, how comfortable are you in managing patients with respiratory complaints?

1Not comfortable 2  somehow comfortable 3 comfortable 4  very comfortable 5 extremely comfortable

4.3. How comfortable are you in diagnosing and managing the following respiratory diseases. Choose the top five diseases which you are most comfortable with, by writing the corresponding figure whereby 1=most comfortable and 5=least comfortable.

| Acute respiratory infections (ARI) |  | Chronic bronchitis |  | Foreign body aspiration |  |
| --- | --- | --- | --- | --- | --- |
| Upper respiratory Tract Infections (URTI) |  | Asthma |  | Bronchiectasis |  |
| Lower Respiratory Tract Infections (LRTI) |  | Lung cancer |  | Pulmonary embolism |  |
| Pneumonia |  | Chronic Obstructive Pulmonary Disease |  | Acute respiratory distress syndrome |  |
| Tuberculosis |  | Bronchiolitis |  | Pulmonary hypertension |  |
| Acute bronchitis |  | Pneumothorax |  | Others (specify) |  |

- 1. On a scale of 1-5, how comfortable are you in taking history and performing a complete physical exam on patients with respiratory complaints?

1Not comfortable 2 somehow comfortable 3 comfortable 4  very comfortable 5 extremely comfortable

- 1. Which of the following procedures are you comfortable performing in a clinical setting? (Check all that apply)

| Pulse oximetry (measuring peripheral oxygen saturation) |  | Nebulization |  |
| --- | --- | --- | --- |
| Reading chest x-rays |  | Demonstrating how to use a spacer |  |
| Interpreting spirometry results |  | Giving oxygen to patients |  |
| Peak flow rate measurement |  | Pulmonary rehabilitation |  |
| Throat swab |  | Chest ultrasound |  |
| Performing and interpreting TB skin test |  | Chest tube insertion |  |

- 1. Which of the following services and equipment do you have at your health facility? (check all that apply)

| X-ray |  | Nebulizers |  |
| --- | --- | --- | --- |
| Spirometry |  | Pulse oximeters |  |
| Sputum smear for typical bacteria |  | Spacers |  |
| Sputum smear for Mycobacteria TB |  | Oxygen therapy equipment |  |
| Mantoux skin test |  | Chest ultrasound |  |
| GeneXpert |  | Chest tube insertion |  |

**5.0. Patient care**

5.1. In your day-to-day clinical practice, which of the following aspects of patient care do you regularly perform? (Check all that apply)

| 1 | Establishing a relationship with patients |  |
| --- | --- | --- |
| 2 | Assessing a patient’s clinical needs |  |
| 3 | Treating patients |  |
| 4 | Planning and organizing individual patient’s care |  |
| 5 | Evaluating patient’s psychological and social needs |  |
| 6 | Giving information to patients and or family caregivers |  |
| 7 | Doing paperwork and/or routine data inputting |  |

**6.0. Team-based approach to patient care**

6.1. At your health facility, do you manage patients as a team?

Yes No

6.2. In the past three years, have you had any training on team-based approach to patient care?

Yes No

6.3. The list below shows some of the aspects of team approach to patient care. Choose the top five aspects that are most important to you starting with the most important =1, and least important=5.

| Mutual trust |  |
| --- | --- |
| Clear roles and division of labour |  |
| Effective communication |  |
| Feedback |  |
| Sharing responsibilities |  |
| Opportunity for mentorship from senior colleagues |  |

6.4. In your health facility, who do you consult in case you encounter a challenging case with respiratory symptoms? Write down all the people you consult. Use designation (e.g. Medical officers, Physician, Paediatrician, Clinical officer) and NOT their names.

1…………………………………………………….

2…………………………………………………….

3……………………………………………………

4…………………………………………………….

5……………………………………………………..

**7.0. Patient education**

7.1. On a scale of 1-5, how often do you provide health education on respiratory diseases?

1Never 2  occasionally 3 sometimes 4  most of the time 5 all the time

7.2. On a scale of 1-5, how comfortable are you in providing health education on respiratory diseases to your patients?

1Not comfortable 2  somehow comfortable 3 comfortable 4  very comfortable 5 Extremely comfortable

**8.0. Patient outcomes**

8.1. At your health facility, do you have any system of measuring patient outcomes?

Yes No

8.2. At your health facility do you have any of the following services to monitor patient outcomes (check all that apply)?

| 1 | Electronic database |  |
| --- | --- | --- |
| 2 | Weekly/monthly reports |  |
| 3 | Periodic surveys |  |
| 4 | Clinical audits |  |
| 5 | Operational research |  |
| 6 | Quality improvement meetings |  |
| 7 | Other |  |

**9.0. Referral**

9.1. Which of the following cases do you usually refer? (Check all that apply)

| Acute respiratory infections (ARI) |  | Chronic bronchitis |  | Foreign body aspiration |  |
| --- | --- | --- | --- | --- | --- |
| Upper respiratory Tract Infections (URTI) |  | Asthma |  | Bronchiectasis |  |
| Lower Respiratory Tract Infections (LRTI) |  | Lung cancer |  | Pulmonary embolism |  |
| Pneumonia |  | Chronic Obstructive Pulmonary Disease |  | Acute respiratory distress syndrome |  |
| Tuberculosis |  | Bronchiolitis |  | Pulmonary hypertension |  |
| Acute bronchitis |  | Pneumothorax |  | Others (specify) |  |

**10.0 Barriers to respiratory care**

10.1. In your opinion, what are some of the barriers and challenges in providing high quality respiratory care to your patients?

………………………………………………………………………………………………………………………………………………………………………………………………………………………………………………………………………………………………………………………………………………………………………………………………………………………………………………………………………………………………………………………………………………………………………………………………………………………………………………………………………………………………………………………………………………………………………………………………………………………………………………………………………………………………………………………………………………………………………

**SECTION B: TRAINING NEEDS**

1. Looking back at your 1^st^ year of clinical practice, do you agree that the training you received sufficiently prepared you to manage patients with respiratory symptoms?

Yes No

1. On a scale of 1-5, how important is it for you to receive further training in respiratory medicine?

1Not important 2 slightly important 3 moderately important

4  very important 5 extremely important

4.0. On a scale of 1-3, in which of the following areas of respiratory medicine do you feel that you need further training?

| **Area** | **No need** | **Somehow need** | **Highly needed** |
| --- | --- | --- | --- |
| ***Diseases*** | | | |
| Asthma |  |  |  |
| COPD |  |  |  |
| Pneumonia |  |  |  |
| Tuberculosis |  |  |  |
| Bronchitis |  |  |  |
| Pulmonary embolism |  |  |  |
| Lung cancer screening |  |  |  |
| Bronchiolitis |  |  |  |
| Acute respiratory distress syndrome |  |  |  |
| Bronchiectasis |  |  |  |
| Pulmonary hypertension |  |  |  |
| Respiratory zoonoses |  |  |  |
| Others (specify) |  |  |  |
| ***Procedures*** | | | |
| History taking and physical examination |  |  |  |
| Ordering tests |  |  |  |
| Interpreting test results |  |  |  |
| Pulmonary rehabilitation |  |  |  |
| ***Others*** |  |  |  |
| Respiratory pharmacology | | | |
| Communication skills |  |  |  |
| Professionalism |  |  |  |
| Care coordination |  |  |  |

1. In your opinion, which of the following course delivery models will be most appropriate for you? (Check only one)
2. **Intensive course:** This will take 10-14 working days and will be conducted at Makerere Lung Institute. Thereafter, the trainees go back to their work stations and start applying the knowledge and skills learnt.
3. **Hybrid course:** This is a combination of an intensive phase of about 3-5 days at Makerere Lung Institute. The trainees will then return to their work station with a package of additional reading material. The trainee will be expected to use the material learned during the intensive phase, and that from self-directed reading to apply the knowledge and skills in their day-to-day work. During this time, they will be mentored by teams from Makerere Lung Institute through on-site visits and telephone/e-mails. After completing the module, the trainees will return to Makerere Lung Institute for sharing experiences and challenges with colleagues. Areas that need clarification will also be addressed.

**Thank you for taking your time to participate in this survey**
