# Supplementary material for: Training needs for Ugandan primary care health workers in management of respiratory diseases: a cross sectional survey
Source: BMC Health Serv Res. 2020 May 11;20:402. doi: 10.1186/s12913-020-05135-3 (PMC7212561; doi:10.1186/s12913-020-05135-3)
Supplement: Supplementary file 2 — Additional file 2. Survey tool for Specialists: Questinnaire on the views of specialists on the need for an Integrated Respiratory Medicine Training Programme for Primary Care Health Workers in Uganda [file 12913_2020_5135_MOESM2_ESM.docx]

**INTEGRATED RESPIRATORY MEDICINE TRAINING PROGRAMME FOR HEALTHWORKERS IN PRIMARY CARE SETTINGS (iBreath project)**

**MAKERERE UNIVERSITY LUNG INSTITUTE COLLEGE OF HEALTH SCIENCES, MAKERERE UNIVERSITY**

**Opinion of Specialists**

In Uganda, respiratory diseases are among the leading causes of death in both children and adults. Until recently, much of the health programmes on lung health focus on communicable diseases such as acute respiratory infections (ARI), pneumonia and tuberculosis. However, there is an emerging yet under-recognized epidemic of non-communicable lung diseases such as asthma, Chronic Obstructive Pulmonary Disease (COPD) and Lung Cancer. The combination of the communicable and non-communicable lung epidemics is a major threat to public health. Unfortunately, there are very few primary care HWs, with the knowledge and skills to effectively manage these epidemics. In recognition of these challenges, Makerere University Lung Institute (MLI) is planning to start an integrated respiratory medicine programme for frontline health care providers.

As a specialist, your views on whether this programme is needed, what it should cover and how it should be organized are important. We are therefore seeking your opinion by participating in this needs assessment survey. Your participation in this survey is voluntary, and the information obtained will be kept anonymous and confidential.

- 1. **Demographics**

Sex Male Female

- 1. Specialty

1. Internist

b) Paediatrician

c) Pulmonologist

- 1. Years of practice in this specialty……………………..

**2.0. Burden of respiratory diseases burden in the health facilities**

In order to provide training relevant content, we need to understand the current burden of respiratory diseases in the various health care facilities.

- 1. In your opinion, what is the proportion of patients that are diagnosed with respiratory diseases in your facility/ward/unit per month?

<20% 20-40%  40-60%  60-80%  >80%

- 1. Below is a list of the common respiratory diagnoses. Choose the top five diagnoses that you think contribute significantly to the burden of respiratory diseases in the population, starting with the most common to the least common ( 1=most common and 5=least common).

| Upper respiratory Tract Infections (URTI) |  | Asthma |  | Bronchiectasis |  |
| --- | --- | --- | --- | --- | --- |
| Lower Respiratory Tract Infections (LRTI) |  | Lung cancer |  | Pulmonary embolism |  |
| Pneumonia |  | Chronic Obstructive Pulmonary Disease |  | Acute respiratory distress syndrome |  |
| Tuberculosis |  | Bronchiolitis |  | Pulmonary hypertension |  |
| Pneumothorax |  | Chronic bronchitis |  | Foreign body aspiration |  |
| Acute bronchitis |  | Others (specify) |  |  |  |

**3.0. Referrals**

3.1. On a scale of 1-5, how often do you see patients that have been referred because of respiratory complaints?

1Never 2 Seldom 3 Sometimes 4  often 5 Almost always

3.2. On a scale of 1-5, how often do you see patients that have been referred to you because of respiratory complaints who you think would otherwise have been managed in the primary care settings?

1Never 2 Seldom 3 Sometimes 4  often 5 Almost always

3.3. In your opinion, what respiratory diseases/complications are frequently referred to you, but you think should be managed appropriately at primary care level.

**4.0. Training needs**

The proposed course will target frontline health care providers in primary care settings including medical officers, clinical officers, and nurses/midwives who are involved in clinical care. The course aims to provide the healthcare workers with knowledge, skills and competences to effectively screen, diagnose and manage patients with respiratory diseases in primary care settings. It will also equip the health workers with aspects such as team approach to patient care, patient-centered care, professionalism and, diagnostics like spirometry and chest x-ray reading. Such aspects are crucial in ensuring good outcomes of patients with respiratory diseases. It will be a delivered as independent modules such that a trainee enrolls for only those courses that are of interest to him/her.

4.1. On a scale of 1-5, how beneficial will a course on respiratory medicine be beneficial to clinicians in primary care settings?

1Not at all 2  slightly beneficial 3 Moderately beneficial 4  Very beneficial 5 extremely beneficial

4.2. In your opinion what knowledge, skills and competencies do primary care health workers need in order to improve access and quality of care for patients presenting with respiratory symptoms?

………………………………………………………………………………………………………………………………………………………………………………………………………………………………………………………………………………………………………………………………………………………………………………………………………………………………………………………………………………………………………………………………………

4.3. Do you think there will be some benefits to the specialist following training healthcare workers in primary settings on respiratory medicine?

Yes No

If Yes, in which way?

……………………………………………………………………………………………………………………………………………………………………………………………………………………………………………………………………………………………………………………………………………………………………………………………………………………………………………………………………………………………………………………………………

4.4. In your opinion, which of the following course delivery models will be most appropriate method of delivery for your staff?

1. **Intensive course:** This will take 10-14 working days and will be conducted at Makerere Lung Institute. Thereafter, the trainees go back to their work stations and start applying the knowledge and skills learnt.
2. **Hybrid course:** This is a combination of an intensive phase of about 3-5 days at Makerere Lung Institute. The trainees will then return to their work station with a package of additional reading material. The trainee will be expected to use the material learned during the intensive phase, and that from self-directed reading to apply the knowledge and skills in their day-to-day work. During this time, they will be mentored by teams from Makerere Lung Institute through on-site visits and telephone/e-mails. After completing the module, the trainees will return to Makerere Lung Institute for sharing experiences and challenges with colleagues. Areas that need clarification will also be addressed.

**5.0. Barriers to quality respiratory care**

5.1. In your opinion, what are the barriers to providing high quality respiratory care in the health care facilities at each of the following levels?

1. Health care providers

………………………………………………………………………………………………………………………………………………………………………………………………………………………………………………………………………………………………………………………………………………………………………………………………………………………………………………………………………………………………

1. Health system

………………………………………………………………………………………………………………………………………………………………………………………………………………………………………………………………………………………………………………………………………………………………………………………………………………………………………………………………………………………………

1. Patients

………………………………………………………………………………………………………………………………………………………………………………………………………………………………………………………………………………………………………………………………………………………………………………………………………………………………………………………………………………………………

**Thank you so much for participating in this survey**
