# Supplementary material for: Training needs for Ugandan primary care health workers in management of respiratory diseases: a cross sectional survey
Source: BMC Health Serv Res. 2020 May 11;20:402. doi: 10.1186/s12913-020-05135-3 (PMC7212561; doi:10.1186/s12913-020-05135-3)
Supplement: Supplementary file 3 — Additional file 3. Survey tool for Policy Makers and Administrators: Questinnaire on the views of on the Policy Makers and Administrators ed. for an Integrated Respiratory Medicine Training Programme for Primary Care Health Workers in Uganda [file 12913_2020_5135_MOESM3_ESM.doc]

**INTEGRATED RESPIRATORY MEDICINE TRAINING PROGRAMME FOR HEALTH WORKERS IN PRIMARY CARE SETTINGS (iBreath project)**

**MAKERERE UNIVERSITY LUNG INSTITUTE COLLEGE OF HEALTH SCIENCES, MAKERERE UNIVERSITY**

**Opinion of Administrators and Policymakers**

In Uganda, respiratory diseases are among the leading causes of death in both children and adults. Until recently, much of the health programmes on lung health focus on communicable diseases such as acute respiratory infections (ARI), pneumonia and tuberculosis. However, there is an emerging yet under-recognized epidemic of non-communicable lung diseases such as asthma, Chronic Obstructive Pulmonary Disease (COPD) and Lung Cancer. The combination of the communicable and non-communicable lung epidemics is a major threat to public health. Unfortunately, there are very few primary care HWs, with the knowledge and skills to effectively manage these epidemics. In recognition of these challenges, Makerere University Lung Institute (MLI) is planning to start an integrated respiratory medicine programme for frontline health care providers.

As an administrator/policymaker, your views on whether this programme is needed, what it should cover and how it should be organized are important. We are therefore seeking your opinion by participating in this needs assessment survey. Your participation in this survey is voluntary, and the information obtained will be kept anonymous and confidential.

**SECTION A**

- 1. **Demographics**
  2. **Sex** Male Female
  3. **Cadre**

| Medical officer |  | Degree midwife |  |
| --- | --- | --- | --- |
| Clinical officer |  | Registered midwife |  |
| Degree Nurse |  | Enrolled midwife |  |
| Registered Nurse |  | Other (Specify) |  |
| Enrolled Nurse |  |  |  |

- 1. Current position of responsibility……………………………………………
  2. Years in service in your current duty station……………………………….
  3. Years in service in previous duty stations…………………………..

**2.0. Burden of respiratory diseases burden in the health facilities**

In order to provide training relevant content, we need to understand the current burden of respiratory diseases in the various health care facilities.

- 1. In your opinion, what is the proportion of patients that are diagnosed with respiratory diseases in the various health facilities every month?

<20% 20-40% 40-60% 60-80%  >80%

- 1. Below is a list of the common respiratory diagnoses. Choose the top five diagnoses that you think contribute significantly to the burden of respiratory diseases in the population, starting with the most common to the least common ( 1=most common and 5=least common).

| Upper respiratory Tract Infections (URTI) |  | Asthma |  | Bronchiectasis |  |
| --- | --- | --- | --- | --- | --- |
| Lower Respiratory Tract Infections (LRTI) |  | Lung cancer |  | Pulmonary embolism |  |
| Pneumonia |  | Chronic Obstructive Pulmonary Disease |  | Acute respiratory distress syndrome |  |
| Tuberculosis |  | Bronchiolitis |  | Foreign body aspiration |  |
| Pneumothorax |  | Chronic bronchitis |  |  |  |
| Acute bronchitis |  | Others (specify) |  |  |  |

**3.0. In-service training**

3.1. Do you provide your staff with opportunities for in-service training?

Yes  No

**If Yes,**

3.2. Did any of the training in the last three (3) years focus on any of the following areas? (Check all that applies).

| 1 | Common infectious diseases e.g. malaria, HIV |  |
| --- | --- | --- |
| 2 | Non-communicable diseases e.g. hypertension, diabetes |  |
|  | Respiratory diseases |  |
| 3 | Maternal Health and newborn care |  |
| 4 | Quality of healthcare |  |
| 5 | Immunization |  |
| 6 | Quality improvement |  |
| 7 | Professionalism |  |
| 8 | Chronic disease management |  |
| 9 | Team-based approach to patient care |  |
| 10 | Patient-centered care |  |
| 11 | Diagnostics procedures for respiratory diseases |  |
| 12 | No in-service training at all |  |

3.3. What kind of learning resources are available and can be accessed by the staff in your facility? (Check all that apply)

Library

Websites

Job aids

**4.0. Training needs**

4.1. On a scale of 1-5, how important is the training of your staff in respiratory medicine?

1Not important 2 slightly important 3 moderately important

4  very important 5 extremely important

4.2. Below are some of the proposed areas for training health workers in respiratory medicine in primary care settings. On a scale of 1-3, in which of the following areas of respiratory medicine would your staff need further training? (1=no need 2=somehow needed 3=highly needed)

| **Area** | **No need** | **Somehow need** | **Highly needed** |
| --- | --- | --- | --- |
| ***Diseases*** | | | |
| Asthma |  |  |  |
| COPD |  |  |  |
| Pneumonia |  |  |  |
| Tuberculosis |  |  |  |
| Bronchitis |  |  |  |
| Pulmonary embolism |  |  |  |
| Lung cancer screening |  |  |  |
| Bronchiolitis |  |  |  |
| Acute respiratory distress syndrome |  |  |  |
| Bronchiectasis |  |  |  |
| Pulmonary hypertension |  |  |  |
| Respiratory zoonoses |  |  |  |
| Others (specify) |  |  |  |
| ***Procedures*** | | | |
| History taking and physical examination |  |  |  |
| Ordering tests |  |  |  |
| Interpreting test results |  |  |  |
| Respiratory pharmacology |  |  |  |
| ***Others*** | | | |
| Communication skills |  |  |  |
| Professionalism |  |  |  |
| Care coordination |  |  |  |

4.3. In your opinion, which of the following course delivery models will be most appropriate method of delivery for your staff?

1. **Intensive course:** This will take 10-14 working days and will be conducted at Makerere Lung Institute. Thereafter, the trainees go back to their work stations and start applying the knowledge and skills learnt.
2. **Hybrid course:** This is a combination of an intensive phase of about 3-5 days at Makerere Lung Institute. The trainees will then return to their work station with a package of additional reading material. The trainee will be expected to use the material learned during the intensive phase, and that from self-directed reading to apply the knowledge and skills in their day-to-day work. During this time, they will be mentored by teams from Makerere Lung Institute through on-site visits and telephone/e-mails. After completing the module, the trainees will return to Makerere Lung Institute for sharing experiences and challenges with colleagues. Areas that need clarification will also be addressed.

**5.0. Barriers to quality respiratory care**

5.1. In your opinion, what are the barriers to providing high quality respiratory care in the health care facilities at each of the following levels?

1. Health care providers

………………………………………………………………………………………………………………………………………………………………………………………………………………………………………………………………………………………………………………………………………………………………………………………………………………………………………………………………………………………………

1. Health system

………………………………………………………………………………………………………………………………………………………………………………………………………………………………………………………………………………………………………………………………………………………………………………………………………………………………………………………………………………………………

1. Patients

……………………………………………………………………………………………………………………………………………………………………………………………………………………………………………………………………………………………………………………………………………………………………………………………………………………………………………………………………………………………

**6.0. Sustainability**

6.1. After training, what kind of support will the trained health workers need to further strengthen and consolidate their knowledge, skills and competencies in providing respiratory care?

…………………………………………………………………………………………………………………………………………………………………………………………………………………………………………………………………………………………………………………………………………………………………………………………

**Thank you so much for participating in this survey**
